# Comparison of Traditional Chinese Manual Therapy (Tuina) and Extracorporeal Shock Wave Therapy in Patients With Nonspecific Neck Pain: Protocol for a Randomized Controlled Trial

**DOI:** 10.2196/96555

**Published:** 2026-07-20

**Authors:** Hongyu Li, Lili Zhao, Zhenning Zhao, Jing Mu, Huisheng Ma

**Affiliations:** 1 Department of Rehabilitation Medicine, General Hospital of Ningxia Medical University, Yinchuan, China; 2 College of Traditional Chinese Medicine, Ningxia Medical University, 1160 Shengli Street, Xingqing District, Yinchuan, 750003, China; 3 Key Laboratory of Dryness Syndrome in Chinese Medicine, Ministry of Education, Ningxia Medical University, Yinchuan, China; 4 Ningxia Regional Key Laboratory of Integrated Traditional Chinese and Western Medicine for Prevention and Treatment of Regional High Incidence Disease, Ningxia Medical University, Yinchuan, China

**Keywords:** Tuina, shock wave therapy, nonspecific neck pain, randomized controlled trial, protocol

## Abstract

**Background:**

Nonspecific neck pain (NNP) is a highly prevalent musculoskeletal condition worldwide, yet optimal noninvasive treatments remain to be established. Tuina and extracorporeal shock wave therapy (ESWT) are both commonly used in clinical practice, but comparative evidence is limited.

**Objective:**

This study aims to compare the clinical efficacy of Tuina versus ESWT in patients with NNP.

**Methods:**

In this randomized, controlled, single-blind trial, 58 patients with NNP will be allocated in a 1:1 ratio into 2 groups: Tuina therapy and ESWT. The Tuina group will receive 30-minute sessions 3 times per week for 4 weeks (12 sessions total). The ESWT group will receive interventions on days 1, 5, 10, 15, 20, 25, and 30 (7 sessions total). The primary outcome measure will be pain intensity measured by the visual analog scale. Secondary outcome measures will include the Neck Disability Index, cervical active range of motion, shear wave elastography (SWE) using musculoskeletal ultrasound, and serum inflammatory factors. All outcome measures will be assessed at baseline, during treatment (2 and 4 wk), and at the 8-week follow-up, except for SWE and inflammatory factors. SWE will be measured at baseline, 4 weeks, and 8-week follow-up; inflammatory factors will be measured at baseline and 4 weeks.

**Results:**

Recruitment began on January 4, 2026, and is expected to be completed by August 2026. As of March 30, 2026, of the planned 58 participants, 26 had been enrolled. Data collection, including follow-up, is projected to finish by October 2026. Data analysis will commence after data collection is completed, and the results are anticipated to be submitted for publication in early 2027.

**Conclusions:**

This trial will provide direct comparative evidence to determine whether Tuina is superior to ESWT for reducing pain and disability in NNP, thereby informing evidence-based clinical decisions. Key design limitations include the inability to blind participants, which we mitigated by using blinded outcome assessors and rigorous allocation concealment.

## Introduction

Neck pain is defined as an unpleasant sensory and emotional experience associated with actual or potential tissue damage, located in the anatomical region extending from the superior nuchal line to the level of the scapular spine [1]. It ranks among the top 5 chronic conditions for prevalence and years lived with disability and represents a substantial individual and socioeconomic burden. Global estimates for 2019 recorded 222.7 million prevalent cases and 22.1 million years lived with disability [2-5]. It is most common in middle age, and women are more affected than men [6].

Most neck pain cannot be attributed to a specific pathoanatomical cause and is therefore classified as nonspecific neck pain (NNP) [7,8]. When pain persists for at least 3 months in the absence of systemic disease, it is termed chronic NNP, which is typically driven by postural and mechanical factors [9,10]. The 1-year incidence of NNP is estimated at 30% to 50%, and approximately 20% of cases become chronic, mainly affecting young adults; consequently, early diagnosis and intervention are critical [11].

**Figure 1.**
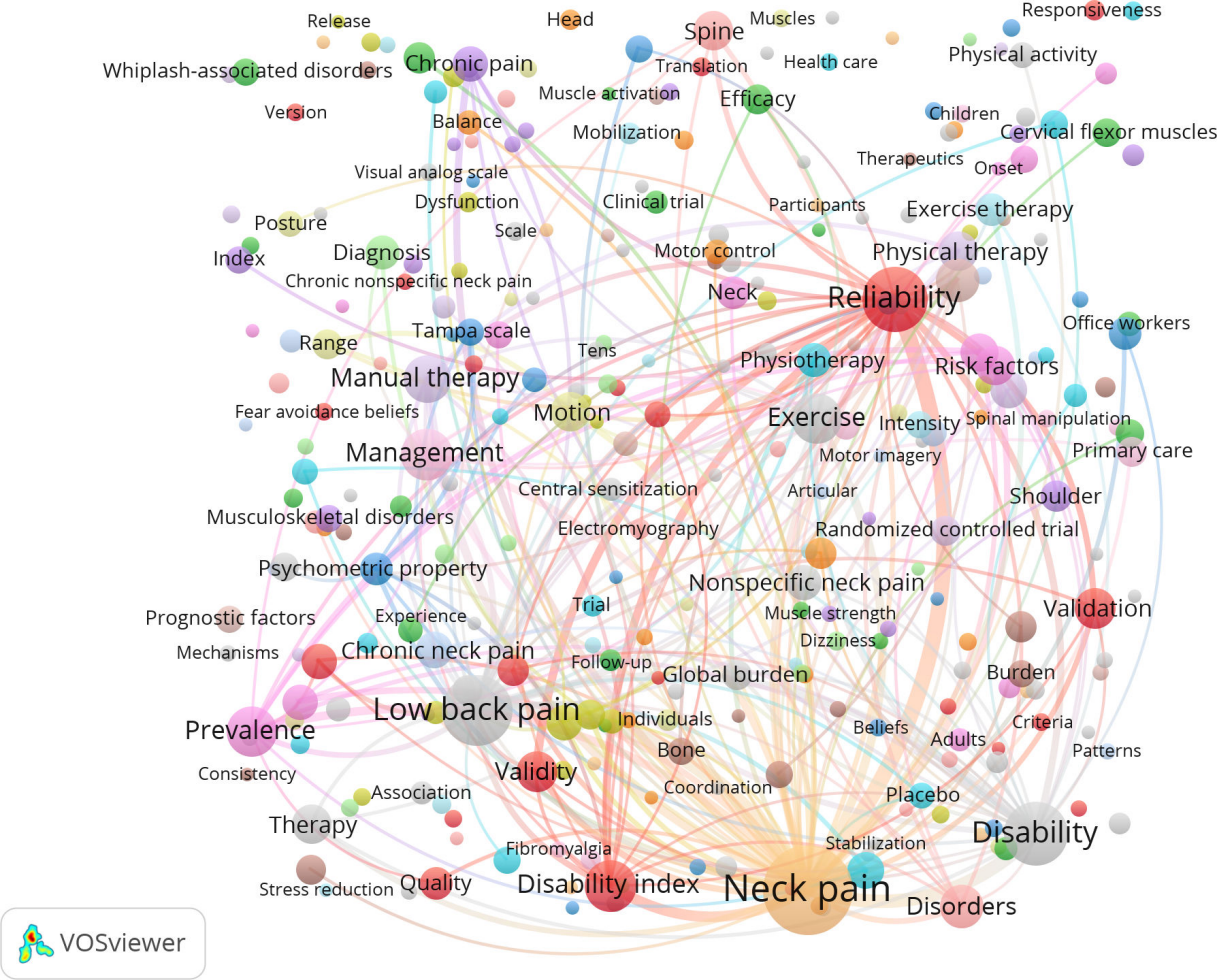
Keywords co-occurrence network of published studies on nonspecific neck pain, visualizing thematic clusters in the research field.

A wide range of pharmacologic and nonpharmacological interventions has been recommended for neck pain; however, systematic reviews report only modest or inconsistent benefits, and concerns about adverse effects limit the use of medications such as nonsteroidal anti-inflammatory drugs [10,12-15]. Based on our preliminary literature review and visualization analysis, manual therapy was identified as one of the top 10 keywords, as shown in Figure 1.

Research shows that Tuina therapy combined with Yijinjing exercise in patients with NNP has a certain clinical effect on pain, functional recovery, and anxiety [16]. For patients with chronic neck pain, it is effective, safe, and relatively economical to perform Tuina therapy 6 times within 3 weeks [17]. Separately, extracorporeal shock wave therapy (ESWT) has been used to deactivate myofascial trigger points in the upper trapezius of individuals with NNP, with promising results [18]. However, these 2 modalities have never been directly compared in a randomized trial. Moreover, existing studies are limited by small sample sizes, the absence of blinding, and short follow-up, leaving the comparative effectiveness and the underlying mechanisms—such as effects on muscle elasticity and systemic inflammation—largely unexplored.

We therefore hypothesized that Tuina therapy would be superior to ESWT in reducing pain intensity and neck-related disability and that both interventions would improve cervical range of motion and muscle elasticity and downregulate serum inflammatory biomarkers. To test this hypothesis, this randomized controlled trial aimed to evaluate the superiority of Tuina therapy over ESWT for NNP. The specific objectives are (1) to compare the 2 interventions in terms of improvements in pain, disability, cervical range of motion, and muscle elasticity and (2) to evaluate their effects on chronic inflammation by measuring the expression of relevant factors in patients’ serum before and after the intervention.

## Methods

### Ethical Considerations

All trial procedures involved in this study were approved by the medical research ethics review committee of the General Hospital of Ningxia Medical University (approval number KYLL-2025-0083, Yinchuan, China, date: January 24, 2025) and prospectively registered at the International Traditional Medicine Clinical Trial Registration platform (registration number ITMCTR2025001377) [19]. The trial will follow the CONSORT (Consolidated Standards of Reporting Trials) guidelines and fulfill the requirements of the SPIRIT (Standard Protocol Items: Recommendations for Interventional Trials) checklist (Checklist 1). All interested participants will be required to sign the written informed consent form with a clear understanding of the study before participation and clearly understand all the contents and processes of the study.

### Study Design

**Figure 2.**
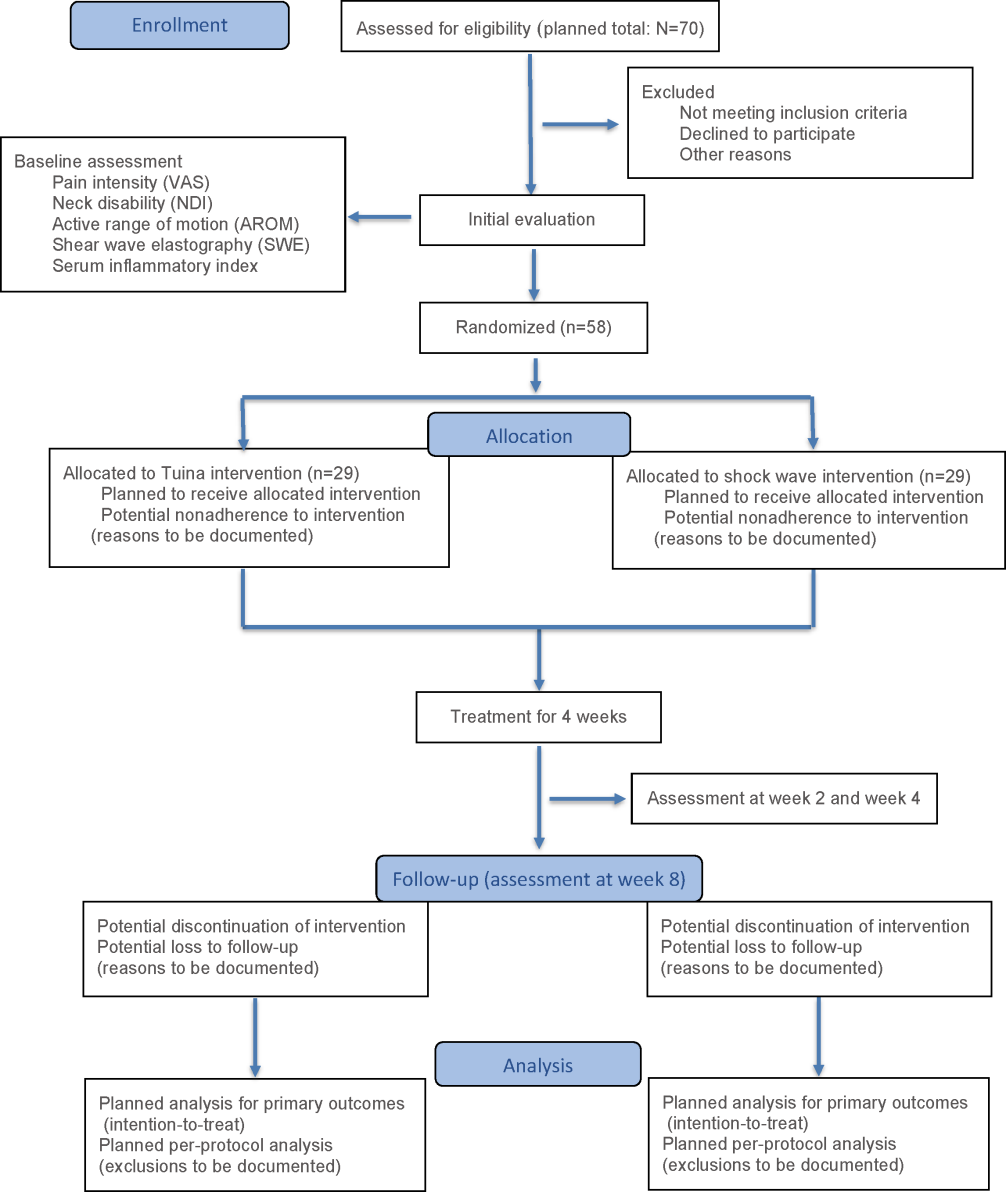
Study flowchart. CONSORT (Consolidated Standards of Reporting Trials) diagram showing participant enrollment, randomization, interventions, and follow-up in a randomized controlled trial of Tuina versus extracorporeal shock wave therapy for nonspecific neck pain. NDI: Neck Disability Index; VAS: visual analog scale.

**Figure 3.**
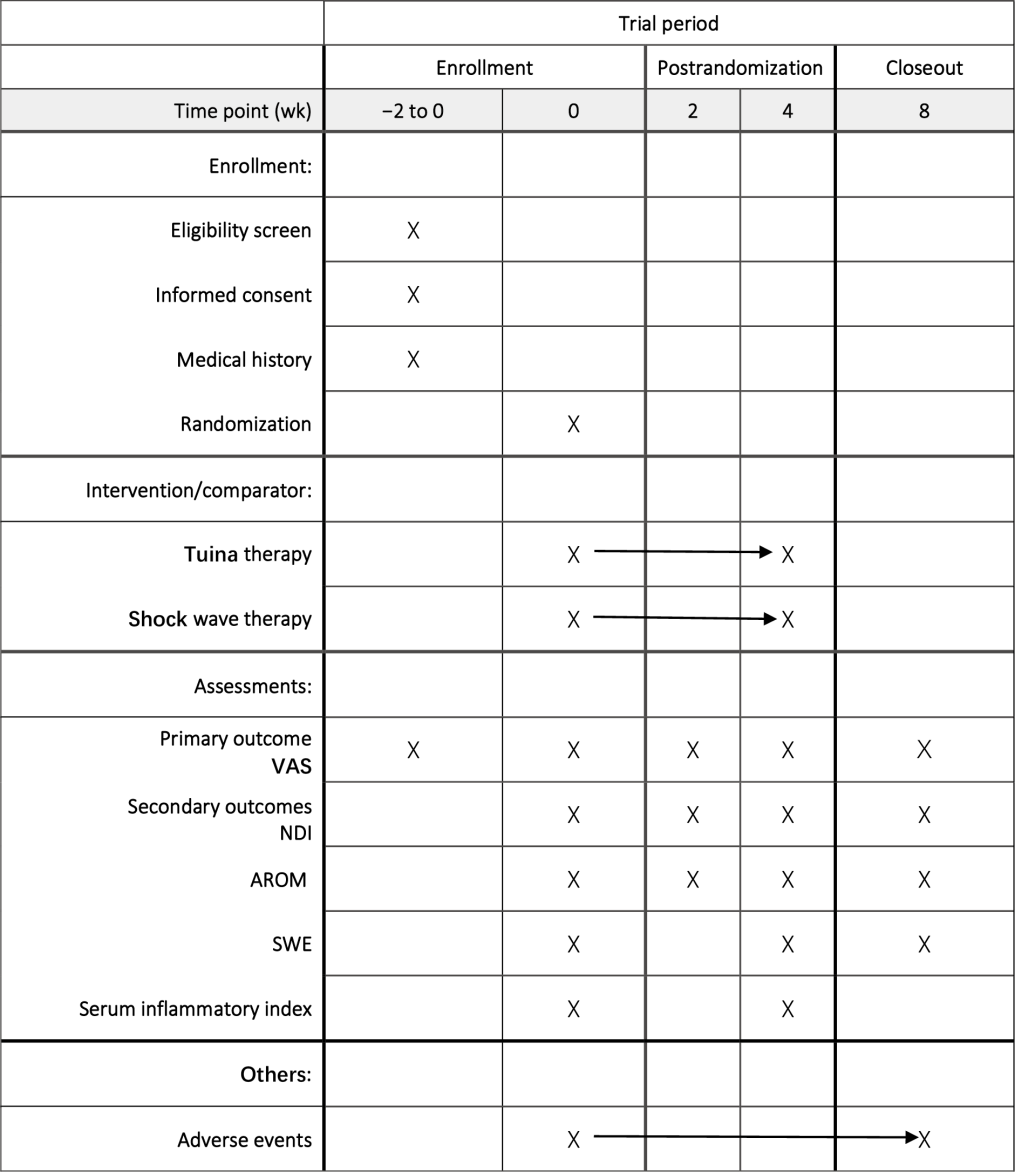
Schedule of enrollment, interventions, and assessments (SPIRIT [Standard Protocol Items: Recommendations for Interventional Trials] schedule) in a randomized controlled trial of Tuina versus extracorporeal shock wave therapy for nonspecific neck pain. AROM: active range of motion; NDI: Neck Disability Index; SWE: shear wave elastography; VAS: visual analog scale.

This is a single-center, randomized, controlled, parallel-group, assessor- and analyst-blinded, superiority trial comparing Tuina therapy versus ESWT in patients with NNP. The trial will be conducted at the General Hospital of Ningxia Medical University. Eligible participants will be randomly assigned in a 1:1 ratio to either the Tuina group or the ESWT group. Both groups will receive their respective intervention in addition to standardized exercise therapy over a 4-week treatment period, followed by a 4-week follow-up (total study duration: 8 wk). The flowchart of the trial process is shown in Figure 2, and the trial period is shown in Figure 3.

Any important protocol modifications will be submitted to the Medical Ethics Committee for approval prior to implementation. Amendments will be updated in the clinical trial registry and communicated to all study personnel through meetings or written notifications. If modifications affect the rights, safety, or willingness of participants to continue, the informed consent form will be updated and participants will be reconsented.

### Recruitment Procedure and Eligibility Criteria

All participants will be recruited through hospital social network media (WeChat) information, hospital posters, and the outpatient department of rehabilitation medicine. According to the diagnostic criteria, inclusion criteria, and exclusion criteria, patients with NNP were screened out.

### Diagnostic Criteria

Diagnosis of NNP was based on the 2014 Canadian evidence-based guidelines for chiropractic therapy in the treatment of adult neck pain and the consensus plan for clinical diagnosis and treatment of NNP through muscle massage (2024 edition) [20,21]: (1) pain located in the anatomical region of the cervical spine; (2) no specific cause such as trauma, infection, tumor, or neuropathy; (3) may or may not be accompanied by headache, restricted cervical motion, or stiffness in one or more directions.

### Inclusion Criteria

Participants will be eligible if they meet all of the following criteria: (1) meet the above diagnostic criteria; (2) age 18 to 60 years old, gender unlimited; (3) 3 points ≤ visual analog scale (VAS) ≤ 5 points; (4) pain duration for more than 3 weeks (ie, beyond the acute phase as defined by the DEGAM S3 [German Society of General Practice and Family Medicine] guideline), which classifies acute neck pain as 0 to 3 weeks [22]; and (5) voluntary participation with signed informed consent.

### Exclusion Criteria

Participants will be excluded if they have any of the following conditions: (1) history of neck trauma, surgery, or tumor; (2) severe cardiac, cerebral, renal, hepatic, or other major organ diseases, mental or behavioral disorders, or inability to cooperate; (3) receipt of Tuina, ESWT, opioid analgesics, antispasmodic analgesics, or anti-inflammatory analgesics within 3 months before enrollment; (4) a confirmed history of infection or familial genetic disease; and (5) pregnant or breastfeeding women.

### Withdrawal Criteria

Participants will be withdrawn if they (1) have incomplete treatment or missing data, (2) experience serious adverse events (SAEs) that prevent further intervention, and (3) show worsening of the condition or are diagnosed with another serious illness during the study period.

### Randomization and Allocation Concealment

A computer-generated random allocation sequence will be prepared by an independent statistician using IBM SPSS 26.0. We will use randomly permuted blocks of varying sizes (2, 4, and 6) to ensure balance between the 2 groups over time and to reduce predictability. A total of 58 subjects will be randomized in a 1:1 ratio to the Tuina group or the ESWT group.

Allocation concealment will be achieved using sequentially numbered, opaque, sealed envelopes. The envelopes will be prepared by an independent statistician who generates the sequence and will be stored in a locked cabinet accessible only to a designated research coordinator who is not involved in participant recruitment, intervention delivery, or outcome assessment. After a participant completes the baseline assessment and signs informed consent, the next sequentially numbered envelope will be opened by the coordinator to reveal the group assignment. The use of envelopes with identical appearances and sequential numbering prevents any foreknowledge of upcoming allocations. Owing to the nature of the interventions, it is not feasible to blind participants or the therapists delivering Tuina and ESWT. This unavoidably introduces the potential for performance bias and expectation bias in self-reported outcomes. To mitigate these risks, we will implement the following measures:

- Blinded outcome assessment: All outcome assessors are blinded to group allocation and are not involved in treatment delivery or data analysis. They will be trained to collect outcomes in a standardized manner and instructed not to discuss treatment with participants.
- Blinded data analysis: Statisticians and data analysts will work with coded data (groups labeled only as “1” and “2”) and will remain blinded until the database is locked and the primary analysis is completed.
- Minimizing differential expectations: Participants in both groups will receive a structured information sheet emphasizing that both interventions are evidence-based and that neither is known to be superior to the other, in order to promote equal treatment credibility.
- Assessment of blinding integrity: At the end of the follow-up period, participants and outcome assessors will be asked to guess group allocation. The proportion of correct guesses will be compared against chance to evaluate the success of blinding procedures for assessors and the effectiveness of expectation management for participants.

After the trial, data will be entered into the database according to subject codes, and the database will be locked after verification. Unblinding will be performed by the project administrator only after all data analyses are completed, at which point the group codes will be revealed.

### Interventions

All interventions will be delivered over a 4-week treatment period, with follow-up assessments conducted 4 weeks after treatment completion. Both groups will receive the same standardized exercise therapy and self-stretching relaxation program. The Tuina group will additionally receive 3-step Tuina therapy, and the ESWT group will additionally receive ESWT. All procedures take place in the treatment room of the Department of Rehabilitation Medicine, General Hospital of Ningxia Medical University. In order to improve the compliance of the subjects, the subjects will receive treatment and related assessment and examination free of charge.

Participants will receive enrollment education on the importance of treatment compliance, appointment reminders 24 hours before each session, flexible scheduling with transportation subsidies, and a treatment log cosigned by the participant and practitioner after each session. Adherence will be monitored through (1) treatment logs cosigned by participant and practitioner after each session, (2) attendance recorded in case report forms, (3) home exercise logs reviewed weekly, and (4) appointment reminders sent 24 hours before each session. Nonadherence thresholds are predefined: participants missing more than 20% of scheduled sessions (ie, ≥3 Tuina sessions or ≥2 ESWT sessions) or completing less than 70% of the prescribed home exercise program will be classified as nonadherent. A per-protocol analysis will be conducted in addition to the intention-to-treat (ITT) analysis. Treatment parameters in the ESWT group will be randomly audited every 2 weeks to ensure protocol consistency.

All interventions will be performed by qualified practitioners. Tuina manipulations will be delivered by practitioners holding a Physician Qualification Certificate and a Physician Practice License in Traditional Chinese Medicine, Acupuncture-Tuina, or Rehabilitation Medicine, with at least 3 years of clinical experience in Tuina. ESWT will be administered by practitioners holding a Physician Qualification Certificate and a Physician Practice License or a nationally recognized rehabilitation therapist certificate, who have completed specialized shock wave training and possess at least 1 year of clinical experience in shock wave therapy. Prior to study initiation, all intervention providers will undergo standardized training on the study protocol and pass a competency assessment. Ongoing quality monitoring will be conducted quarterly throughout the study.

Patients can choose to withdraw from the study at any time. Any significant improvement or deterioration in the condition of a participant, including adverse events (AEs), will be monitored throughout the trial. AEs will be reported to the principal investigator (PI), who will monitor the affected participants and investigate the potential causes.

### Exercise Therapy and Self-Stretching

To ensure comparability, the exercise therapy and self-stretching relaxation training are fully standardized across both groups. The program consists of (1) cervical isometric exercises (flexion, extension, lateral flexion, and rotation, each held for 10 s, 10 repetitions per direction); (2) upper trapezius and levator scapulae self-stretching (30 s per muscle, 3 repetitions each); and (3) postural reeducation. This session is performed at each treatment visit and prescribed as a daily home program. Adherence to the home program is recorded in a self-reported log. Because the exercise component is identical for both groups, any between-group differences observed at the end of the trial can be attributed to the added Tuina or ESWT rather than to the exercise itself. In the primary analysis, group allocation will be the independent variable, and the exercise protocol is considered part of the standard background care that is balanced by randomization.

### Tuina Therapy

The most commonly used methods for the treatment of NNP were loosening tendons, pulling tendons, and smoothing tendons. The treatment was performed 3 times a week for a total of 4 weeks. The operation time and key points of each technique are shown in Table 1.

**Table 1.** Detailed manipulation of Tuina therapy used in the intervention group for patients with nonspecific neck pain in this randomized controlled trial.

Technique name	Frequency	Key points
Loosening tendons	100 times/min, 5 min	Put the thumb and index finger pulp on the muscles on both sides of the cervical spine, from top to bottom, and massage the neck and shoulder muscles circularly.
Pulling tendons	80 times/min, 5 min	The thumb pulp focuses on one side of the neck and shoulder muscles, and the 2 sides are moved alternately.
Smoothing tendons	80 times/min, 5 min	Put the pulp of the thumb and index fingers of both hands under the occipital posterior tuberosity of the neck and push and smooth the neck muscles from top to bottom.

The 3 manipulations are applied with distinct depth and force characteristics:

- Loosening tendons: targets the superficial muscles and subcutaneous tissue, applied with light-to-moderate pressure (approximately 5‐15 N).
- Pulling tendons: reaches the intermediate to deep muscle belly and myofascial attachments, applied with moderate-to-firm pressure (approximately 10‐25 N).
- Smoothing tendons: acts on the superficial to intermediate muscle layers, applied with light-to-moderate pressure (approximately 5‐15 N).

The force ranges are based on published biomechanical data on cervical Tuina. The end point of force application is the patient’s subjective sensation of soreness and distension (deqi-like sensation) without sharp pain. Each session lasts approximately 20 to 30 minutes, including brief rest intervals between techniques.

### Extracorporeal Shock Wave Therapy

ESWT will be performed using a Swiss Storz extracorporeal shock wave therapeutic apparatus (MP50). The MP50 device delivers radial (dispersive) shock waves, characterized by a divergent wave pattern that distributes energy over a broader, more superficial tissue area. Each session will deliver 2000 shocks at a pressure of 1.5 to 2.0 bar and a frequency of 10 to 12 Hz, with moderate handle pressure. Based on the pressure range and radial wave characteristics, the estimated energy flux density ranges from 0.10 to 0.20 mJ/mm², which falls within the medium-energy category as defined by international consensus guidelines (medium estimated energy flux density: 0.08‐0.28 mJ/mm²). The therapy will target tender points and the surrounding soft tissues. Treatment will be administered on days 1, 5, 10, 15, 20, 25, and 30.

### Outcomes

#### Overview

The primary outcome measure will be the VAS. Secondary outcome measures will include the Neck Disability Index (NDI), cervical active range of motion (AROM), shear wave elastography (SWE) using musculoskeletal ultrasound, and serum inflammatory factors. The assessment schedule is as follows: all outcome measures will be evaluated at baseline, during treatment (2 and 4 wk), and at the 8-week follow-up, except for SWE and inflammatory factors. SWE will be measured at baseline, 4 weeks, and follow-up; inflammatory factors will be measured at baseline and 4 weeks.

#### Primary Outcome Measurement

The VAS is a commonly used instrument for pain assessment, designed to enable patients to quantify their pain intensity using a simple linear scale. A 10-centimeter scale will be employed, with end points labeled “0” (no pain) and “10” (the most severe pain imaginable). Patients will indicate the point on the scale that reflects their current pain experience. The primary efficacy end point is the change in VAS score from baseline to the end of the 4-week treatment period. The VAS is primarily utilized to evaluate pain intensity; it may also serve to compare treatment outcomes and to monitor changes in emotional and functional status.

#### Secondary Outcome Measurements

##### Neck Disability Index

The NDI consists of 10 items evaluating 2 domains: neck pain and associated symptoms (pain intensity, headache, concentration, and sleep) and activities of daily living (personal care, lifting, reading, work, driving, and recreation). Each item is rated on a 0 to 5 scale, yielding a maximum total score of 50. The total score is expressed as a percentage, with 0% representing no disability and 100% representing complete disability; higher scores reflect more severe functional impairment.

##### Active Range of Motion

Cervical AROM will be assessed using a goniometer, evaluating 6 directions: flexion, extension, left and right lateral flexion, and left and right rotation. During measurement, the patient will be seated in a relaxed posture with the head and neck in a neutral position. The patient will be asked to actively perform each movement 3 times, and the average value will be used for analysis. Normal reference values for cervical AROM are 30° to 45° for flexion, 30° to 45° for lateral flexion, and 60° to 80° for rotation.

##### Shear Wave Elastography

SWE will be performed using a high-end color Doppler ultrasound system equipped with SWE functionality, using a high-frequency linear array transducer (frequency range: 4‐18 MHz). For the sternocleidomastoid muscle, the patient will be placed in the supine position with the head rotated slightly to the contralateral side to expose the muscle belly. For the upper trapezius muscle, the patient will be seated in an upright position with the head in neutral alignment and arms relaxed at the sides. Both measurements will be performed with the patient instructed to remain relaxed and avoid voluntary muscle contraction. Conventional B-mode ultrasound will be performed first to localize the bilateral sternocleidomastoid muscles and the upper trapezius muscles. The system will be switched to the SWE mode. The transducer will be placed gently on the skin surface (without compression), and stable elastographic images will be acquired along the longitudinal axis of the muscle fibers. After the image stabilizes for 5 seconds, the region of interest will be placed in the central portion of the muscle belly, avoiding blood vessels and bone. Shear wave velocity (m/s) will be measured. Three repeated measurements will be performed at each site, and the mean value will be used for statistical analysis.

##### Serum Inflammatory Factors

Before and after treatment, 4 mL of venous blood will be collected from each patient using a separating gel tube. The collection will be performed by a professional nurse from the Rehabilitation Medical Center of the General Hospital of Ningxia Medical University. Blood samples will be allowed to stand at room temperature for 20 minutes, followed by centrifugation at 3500 rpm for 10 minutes. The upper serum layer will be separated and stored in a refrigerator at −80 °C. Serum levels of stromal cell–derived factor-1, C-X-C chemokine receptor type 4, NOD-like receptor protein 3, interleukin-1β, interleukin-8, interleukin-6, and tumor necrosis factor-α will be measured using enzyme-linked immunosorbent assay.

### Efficacy Evaluation Criteria

The efficacy of the intervention will be evaluated using the improvement rates of the VAS for pain intensity and the NDI for functional status. The percentage improvement for both measures will be calculated as (baseline score−posttreatment score)/baseline score×100%. Clinical response will be classified into 3 categories based on simultaneous satisfaction of both criteria: markedly effective (VAS improvement ≥50% and NDI improvement ≥50%), effective (VAS improvement ≥30% and <50%, NDI improvement ≥30% and <50%), and ineffective (VAS improvement <30% and NDI improvement <30%). If a patient meets different classification levels across the 2 measures, the lower classification will be assigned. The overall clinical effective rate will be calculated as (number of markedly effective cases+number of effective cases)/total number of eligible cases×100%. The threshold of 30% improvement represents the minimum clinically important difference established for both VAS and NDI in patients with neck pain, while the 50% threshold represents substantial clinical benefits, indicating a truly successful outcome [23-25].

### Data Collection and Management

Data will be collected using printed case report forms and entered into an electronic case report form by independent researchers in a timely manner. Paper case report forms will be stored in a locked cabinet in the researcher’s office, and the electronic dataset will be deidentified and locked. Access to these records will be limited to authorized data administrators and statisticians. All study documents will be retained for at least 5 years after publication.

Following the ITT principle, all randomized participants will be included in the final analysis. For participants who discontinue the intervention or deviate from the protocol, primary and secondary outcome data will still be collected at scheduled time points whenever possible, AEs will be continuously monitored, and detailed reasons for discontinuation or deviation will be documented.

### Monitoring and Quality Control

#### Overview

A formal independent data monitoring committee will not be established because both Tuina and ESWT are noninvasive, low-risk interventions with well-established safety profiles, and no interim efficacy or futility analyses are planned. Instead, the PI will assume responsibility for safety monitoring. The PI will review AE data weekly and submit periodic safety summaries to the institutional ethics committee. All SAEs will be reported to the ethics committee within 24 hours of the investigator becoming aware of the event.

As ESWT may cause transient procedural pain or discomfort, participants in the ESWT group will be asked to rate their pain during treatment using VAS after each session. A score ≥7 or any persistent discomfort lasting beyond the treatment session will trigger a review by the PI. If necessary, ESWT parameters (pressure and frequency) will be adjusted, or the session will be terminated early.

#### Study Discontinuation Criteria

The trial may be discontinued prematurely if (1) the ethics committee determines that the risks outweigh the benefits, (2) the PI identifies an unacceptable incidence of treatment-related SAEs, or (3) recruitment is persistently below the expected rate and cannot reach the target sample size within the planned time frame. In the event of study termination, all enrolled participants will be offered the best standard care and followed up according to the protocol.

#### Individual Participant Stopping Rules

The study intervention will be discontinued for an individual participant if (1) the participant withdraws consent, (2) an SAE occurs that is assessed as probably or definitely related to the intervention, (3) the participant experiences intolerable procedure-related pain (ESWT-related pain score ≥7 on 2 consecutive sessions despite parameter adjustment), or (4) the PI determines that continuation poses an unacceptable risk to the participant. Discontinued participants will continue to be followed up for outcome assessment according to the ITT principle.

#### AE Management

AEs will be classified by severity (mild, moderate, and severe) and causality (definitely, probably, possibly related, and unrelated). Participants experiencing moderate or severe AEs will be managed clinically according to standard practice and followed until resolution or stabilization. Any SAE will trigger an immediate suspension of the study intervention for the affected participant and a formal causality assessment by the PI. The participant will be unblinded if necessary for clinical management.

### Sample Size Calculation

The sample size was calculated using G*Power (version 3.1.9.7; Heinrich Heine University Düsseldorf). The primary outcome is the change in VAS score from baseline to week 4. Based on published literature, the minimal clinically important difference for VAS is 1.5 points, with a pooled SD of 1.8 points, yielding an effect size (Cohen *d*) of 0.83 [26,27]. Using a 2-independent-samples *t* test with a 2-sided significance level of *α*=.05 and power (1-β)=0.80, the required sample size is 24 per group (48 in total), with an actual power of 81.2%. Allowing for a 15% dropout rate, the final sample size is set at 29 per group (58 in total).

### Data Analysis

#### Analysis Populations

Three populations will be defined: the ITT population (all randomized, primary analysis), the per-protocol population (≥80% sessions completed and primary outcome assessed, sensitivity analysis), and the safety population (≥1 intervention received, safety analyses).

#### Primary Outcome Analysis

The primary end point is the change from baseline in the VAS score at week 4. The primary analysis will use a mixed-effects model for repeated measures (MMRM) with VAS at weeks 2, 4, and 8 as dependent variables. The model will include the treatment group, time, and their interaction as fixed effects and baseline VAS as a covariate; an unstructured covariance matrix will model within-subject correlations. From this model, the estimated difference between Tuina and ESWT at week 4 will be derived, along with its 95% CI. MMRM inherently accommodates missing data under the missing-at-random assumption. Multiple imputation will be used as a sensitivity analysis to check robustness.

#### Secondary Outcomes and Multiplicity

Key secondary outcomes (NDI, cervical AROM, SWE, and inflammatory biomarkers) will be analyzed using the same MMRM framework, with baseline values as covariates. To control for multiplicity, the Holm-Bonferroni correction will be applied to the key secondary end points measured at week 4. Comparisons at other time points will be considered exploratory and will be reported with unadjusted *P* values and 95% CIs. Categorical responder analyses will be supportive.

#### Baseline and Safety Comparisons

Baseline characteristics will be summarized by the group using descriptive statistics. Continuous variables will be compared using the independent *t* test or Mann-Whitney *U* test as appropriate; categorical variables with the chi-square test or Fisher exact test. Safety end points will be presented descriptively.

All analyses will use 2-tailed tests, and the primary outcome will be tested at *α*=.05. Analyses will be performed using IBM SPSS 26.0 and GraphPad Prism 9.0.

## Results

Recruitment began on January 4, 2026, and is expected to be completed by August 2026. As of March 30, 2026, 26 of the planned 58 participants had been enrolled. Data collection, including follow-up, is projected to finish by October 2026. Data analysis will commence after data collection is completed, and the results are anticipated to be submitted for publication in early 2027.

## Discussion

This randomized controlled trial will provide the first direct comparison of Tuina therapy and ESWT for patients with NNP. While both interventions have individually demonstrated benefits for NNP, no head-to-head trial exists, leaving uncertainty about which modality offers superior clinical outcomes or whether they are equivalent. By integrating clinical end points with muscle elasticity and serum inflammatory biomarkers, this trial also aims to explore the mechanisms through which these distinct therapies exert their effects.

Tuina, a manual therapy grounded in traditional Chinese medicine, and ESWT, a biophysical modality based on mechanotransduction principles, represent 2 fundamentally different therapeutic approaches. Previous studies suggest that Tuina reduces pain and improves function through the mechanical stimulation of soft tissues and the modulation of descending inhibitory pathways, while ESWT promotes tissue repair and attenuates inflammation via acoustic wave–induced mechanotransduction [28-32]. Despite these mechanistic differences, their comparative clinical effectiveness has not been established.

Current evidence for Tuina in individuals with NNP comes from randomized trials and systematic reviews demonstrating improvements in pain and disability. For ESWT, a recent network meta-analysis ranked it second among 6 biophysical agents for neck pain rehabilitation, and meta-analyses have reported large effect sizes for pain reduction in myofascial pain syndromes [33-35]. A randomized controlled trial comparing ESWT with manual therapy in patients with cervicogenic headache found both interventions equally effective in improving pain, neck disability, and muscle stiffness, suggesting that ESWT may serve as a viable alternative to manual therapy [36]. Nevertheless, the absence of trials directly comparing Tuina with ESWT in NNP represents a critical gap, which this protocol addresses.

This trial has several methodological limitations that should be acknowledged. First, owing to the nature of the interventions, participants and therapists cannot be blinded; this may introduce performance and expectation biases, which we have sought to mitigate through blinded outcome assessment, standardized information on treatment credibility, and analysis of blinding integrity. Second, the treatment session frequency differs between groups (12 Tuina sessions vs 7 ESWT sessions). This discrepancy reflects real-world clinical practice but could confound the estimated treatment effect; we plan to examine the influence of session attendance in sensitivity analyses and discuss it as a potential limitation. Third, the sample size (58 participants) was calculated to detect a large effect size; if the true between-group difference is smaller, the trial may be underpowered. Accordingly, treatment effect estimates and CIs will be emphasized over binary significance statements. Fourth, this is a single-center study, which may limit generalizability; a future multicenter trial would enhance external validity. Fifth, the 4-week follow-up period is relatively short and may not capture the long-term sustainability of treatment effects.

The findings of this trial are expected to provide comparative data that may inform clinical decision-making for individuals with NNP. If Tuina proves superior to ESWT in reducing pain and disability, its advantages of low equipment requirements and patient comfort could support its adoption as a preferred nonpharmacological treatment, particularly in settings with limited access to shock wave devices. Conversely, if Tuina does not demonstrate superiority, ESWT would retain its role as an evidence-based physical modality, while Tuina might still be considered a viable alternative, given its practical advantages, provided that its clinical effects are comparable. Regardless of the outcome, the trial will generate evidence on the differential effects of these 2 interventions on pain, function, muscle elasticity, and inflammatory biomarkers, contributing to more individualized treatment selection.

In conclusion, this protocol describes a methodologically rigorous randomized trial designed to address a specific evidence gap. The results, interpreted within the context of the acknowledged limitations, will contribute to the evidence base for nonpharmacological management of NNP.

## Supplementary material

10.2196/96555 Checklist 1 SPIRIT checklist.

## Abbreviations

AE: adverse event
AROM: active range of motion
CONSORT: Consolidated Standards of Reporting Trials
DEGAM: German Society of General Practice and Family Medicine
ESWT: extracorporeal shock wave therapy
ITT: intention-to-treat
MMRM: mixed-effects model for repeated measures
NDI: Neck Disability Index
NNP: nonspecific neck pain
PI: principal investigator
SAE: serious adverse event
SPIRIT: Standard Protocol Items: Recommendations for Interventional Trials
SWE: shear wave elastography
VAS: visual analog scale
